# Looking for a pattern: An MEG study on the abstract mismatch negativity in musicians and nonmusicians

**DOI:** 10.1186/1471-2202-10-42

**Published:** 2009-04-30

**Authors:** Sibylle C Herholz, Claudia Lappe, Christo Pantev

**Affiliations:** 1 Institute for Biomagnetism and Biosignalanalysis, University of Münster, Malmedyweg 15, D-48149 Münster, Germany

## Abstract

**Background:**

The mismatch negativity (MMN) is an early component of event-related potentials/fields, which can be observed in response to violations of regularities in sound sequences. The MMN can be elicited by simple feature (e.g. pitch) deviations in standard oddball paradigms as well as by violations of more complex sequential patterns. By means of magnetoencephalography (MEG) we investigated if a pattern MMN could be elicited based on global rather than local probabilities and if the underlying ability to integrate long sequences of tones is enhanced in musicians compared to nonmusicians.

**Results:**

A pattern MMN was observed in response to violations of a predominant sequential pattern (AAAB) within a standard oddball tone sequence consisting of only two different tones. This pattern MMN was elicited even though the probability of pattern deviants in the sequence was as high as 0.5. Musicians showed more leftward-lateralized pattern MMN responses, which might be due to a stronger specialization of the ability to integrate information in a sequence of tones over a long time range.

**Conclusion:**

The results indicate that auditory grouping and the probability distribution of possible patterns within a sequence influence the expectations about upcoming tones, and that the MMN might also be based on global statistical knowledge instead of a local memory trace. The results also show that auditory grouping based on sequential regularities can occur at a much slower presentation rate than previously presumed, and that probability distributions of possible patterns should be taken into account even for the construction of simple oddball sequences.

## Background

Our acoustical environment contains regularities as well as random noise. In order to predict upcoming events it is important to be able to separate the meaningful patterns from random variations. In order to extract such regular patterns, we need the ability to integrate acoustic information over a period of time. One method for investigating if a certain regularity in a sound sequence has been encoded is measuring neuronal responses to *violations *of this regularity, e.g. the mismatch negativity (MMN). The MMN is an early response of auditory cortex neurons to an unexpected stimulus (a 'deviant') in a sequence of otherwise regular auditory stimuli that occurs approximately 100 to 250 ms after onset of the deviant stimulus[1,2]. For example, in a pitch MMN paradigm, rare tones of a high pitch within a sequence of tones of a low pitch will elicit an MMN response. In most MMN paradigms, stimulus onset asynchronies (SOA) are in the range of less than 200 ms [3] up to several seconds [4,5]. The MMN can be observed even if subjects are not paying attention to the stimulation and even if they cannot detect the deviants behaviorally [6,7], but it can also be modulated by attention [8].

The MMN can be elicited by very simple changes like a pitch difference between standard and deviant stimuli, but also by violations of more complex regularities within a tone sequence. For example, a regularity such as "long tones are followed by high tones" in a sequence composed of tones varying in duration and pitch can be pre-attentively extracted and violations of this rule (e.g., a low tone after a long tone) elicit an MMN even if the subjects are not consciously aware of this regularity [7]. Also, Tervaniemi et al. reported an MMN in response to repeated or ascending tone intervals within series of descending tone intervals [9]. Similarly, occasional lengthening a sequential pattern of tones established by a repeating numerical regularity (AAAABBBBCCCC) by one tone (BBBBB) also elicits an MMN [3]. As such deviants can only be identified based on their pattern violation, but not based on physical properties, the presence of an MMN in response to such a pattern deviant can be seen as an indication of whether the brain has encoded the regularity in the first place. However, not only the presence but also the absence of the MMN can be an indication of underlying processes, e.g. auditory grouping. Sussman and Gumenyuk [10] used a pitch MMN paradigm to investigate auditory grouping of a short sequential pattern (AAAB) which was repeatedly presented. An MMN to B tones (deviating in pitch compared to A tones) was observed using an SOA of 400 ms between individual tones, but not at an SOA of 200 ms, which was interpreted as evidence that auditory grouping of the pattern as a whole only occurs at SOAs shorter than 400 ms.

The memory trace theory of the MMN states that the prediction of upcoming sounds is based on a sensory memory trace that has a restricted temporal capacity. The duration of this memory trace has been estimated to be around 10 s [5]. Thus, in order to explain an MMN within this theory, at least two repetitions of the standard pattern and the deviant pattern would have to occur within the time window that is encompassed by the memory trace in order to detect the violation of the pattern. However, the concept of a local sensory memory trace cannot explain all findings of MMN and has been challenged in several studies. In our previous study [11] we were able to show that an imagery MMN can be elicited by tones that discontinued imagined familiar melodies. Importantly, this imagery MMN was based on the imagined long-term memory representation of the familiar melodies, and not on an abstract regularity within the tone sequence. This finding suggested that the classical sensory memory trace cannot be the only basis for the MMN, but rather that the MMN might represent a more general mechanism of expectancy violation detection, and that the expectation or prediction must not necessarily be based on local regularities. Also, Sussman and colleagues showed for single and double tones that the global context of stimulus probabilities (the probabilities of tones within the whole sequence), and not only the local context (probabilities of tones directly preceding a deviant) determined if a tone was perceived as a deviant and if it elicited an MMN [12,13]. However, to our knowledge, the effects of global probabilities have not been investigated for more complex sequential patterns of tones.

In the present study, we investigated if a pattern MMN might not only be elicited by violations of local regularities but also in response to violations of predictions based on a global regularity that can only be picked up over a longer time range. We constructed a simple tone sequence composed of only two different tones which was strongly reminiscent of a standard pitch oddball sequence, and in which one predominant pattern (the standard pattern AAAB) with a frequency of occurrence of 0.5 was "hidden" among similar, longer patterns (AAAAB, AAAAAB). In previous studies on the MMN to sequential pattern violations [3,14], pattern deviants were very infrequent (10%), and the SOA of the tones was very short (max 195 ms), and therefore an interpretation based on a local memory trace is feasible. Also, the patterns as such were relatively easy to identify based on pitch changes on pattern boundaries. In contrast, in the present study pattern deviants were very frequent (50%), the SOA of tones quite long (1 s) and the pattern did not 'pop out' but had to be extracted based on a longer time range.

By means of magnetoencephalography (MEG) we investigated whether violations of this AAAB pattern would elicit a pattern MMN under these conditions, and if the pattern MMN is lateralized. Also, as the ability to extract regularities from sound sequences is central to the processing of music and as a number of studies using MMN paradigms have shown that this ability is more pronounced in trained musicians [3,14-18] and can be trained in nonmusicians [19], musicians and nonmusicians were compared in the present study in order to investigate the effect of musical expertise on the ability to extract sequential patterns from a global context.

## Results

**Figure 1:**
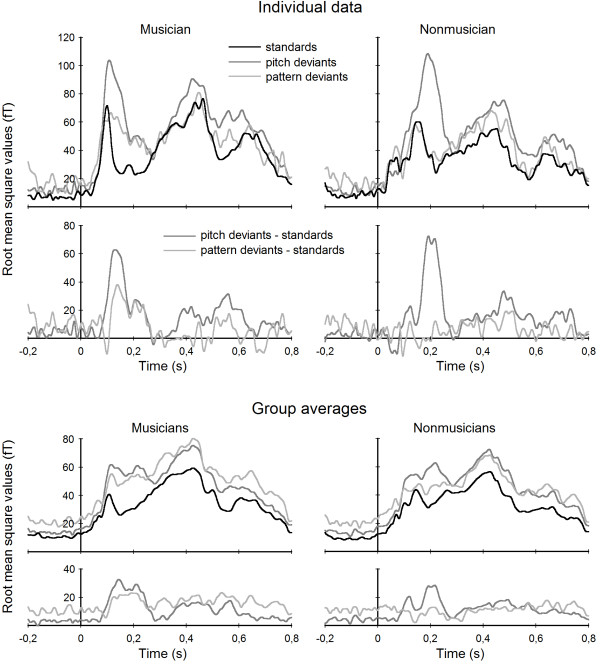
**Root mean square data**. Root mean square values reflecting overall signal strength in response to standards (black), pitch deviants (medium grey) and pattern deviants (light grey), and difference curves (pitch deviants – standards: medium grey; pattern deviants – standards: light grey) showing the MMN responses, for two individual subjects (one musician and one nonmusician, upper panels) and for group averages of musicians and nonmusicians (lower panels).

Figure 1 shows root mean square values of standard and deviant responses and the differences for both pattern and pitch conditions for one musician and one nonmusician and for the group means. Root mean square values reflect overall strength of cortical activation. Although head position and head size are not taken into account, these data can illustrate individual and average responses. Dipoles were then modelled for the pitch MMN responses. Pitch MMN dipole locations (x, y, z, given in cm) of musicians (left hemisphere: 1.85, 3.20, 5.73; right hemisphere: 2.08, -3.58, 5.62) and nonmusicians (left hemisphere: 2.30, 3.66, 5.51; right hemisphere: 2.21, -3.28, 5.70) did not differ between groups (independent t-tests for x, y, and z-axes in both hemispheres; left hemisphere x-axis: t(19) = 1.252, p = .226; y-axis: t(19) = 1.591, p = .128; t(19) = -.383, p = .706; right hemisphere x-axis: t(19) = .292, p = .773; y-axis: t(19) = 1.244, p = .228; z-axis: t(19) = .134, p = .895). The individual localisations of the pitch MMN dipoles were used for the computation of both the pitch and the pattern MMN source waveforms.

**Figure 2:**
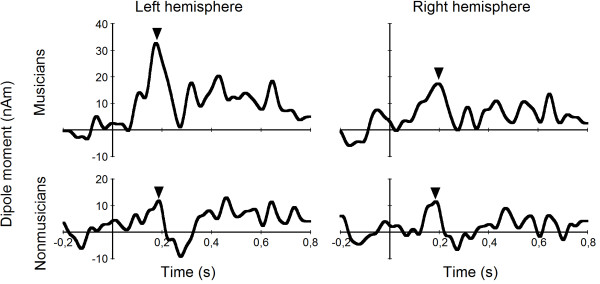
**Pattern MMN in musicians and nonmusicians**. Group averages of the source waveforms showing the pattern MMN (marked by triangles) for musicians and nonmusicians, and for both hemispheres.

Group averages of source waveforms for the pattern MMN for both hemispheres, which were computed from the dipole models, are shown in Figure 2. The pattern MMN is clearly visible in musicians, whereas it is less pronounced in nonmusicians. In the analysis of variance of pattern MMN amplitudes the main effect of group did not reach significance (*F*[1, 19] = 2.411, *p *= .137). However, there was a significant main effect of hemisphere (*F*[1, 19] = 4.837, *p *= .040) and an interaction of group and hemisphere (*F*[1, 19] = 4.591, *p *= .045), indicating that the effect was stronger in the left hemisphere for musicians.

**Figure 3:**
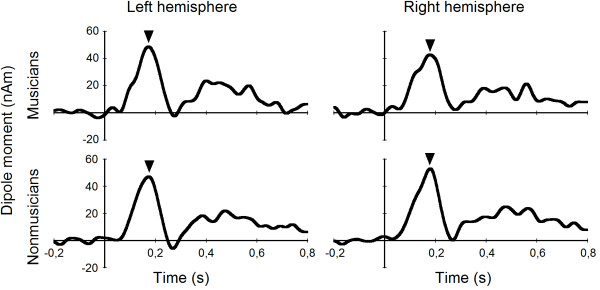
**Pitch MMN in musicians and nonmusicians**. Group averages of the source waveforms showing the pitch MMN (marked by triangles), which was used as a control, for both groups and hemispheres.

As expected, the pitch difference of the two tones elicited a clear MMN response in both groups. Figure 3 shows the responses to the pitch difference in musicians and nonmusicians for both hemispheres. A mixed model analysis of variance with factors group and hemisphere did not yield any main effects (main effect of group *F*[1, 19] = 0.100, *p *= .756; main effect of hemisphere *F* [1, 19] = 0.001, *p *= .973) or interaction (*F*[1, 19] = 2.890, *p *= .105).

**Figure 4:**
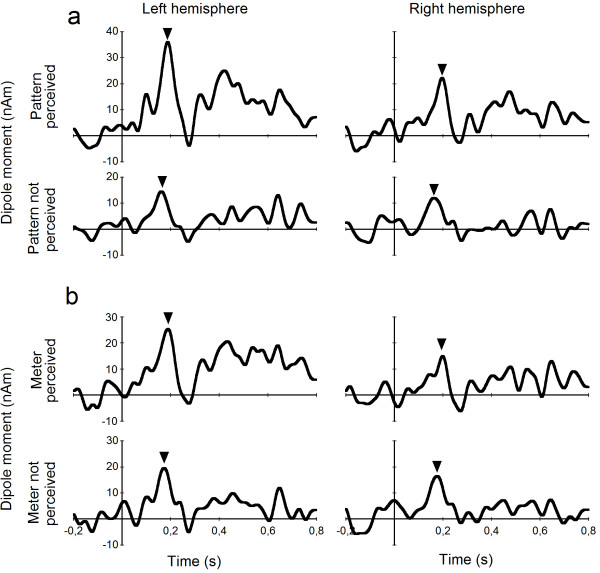
**Pattern MMN grouped by perception of pattern and meter**. Group averages of the pattern MMN (marked by triangles) for both hemispheres for subjects grouped according to perception of the pattern (a) and according to perception of a musical meter within the sequence (b).

Six out of ten musicians but only three out of eleven nonmusicians reported the detection of the prevailing pattern of three lower tones succeeded by a higher tone in the sequence during questioning after the experiment (χ^2 ^= 2.29, p = .13). An analysis of variance of the amplitude of the pattern MMN with correct identification of the pattern as between-subjects factor revealed a significant main effect of group (*F*[1, 19] = 5.589, *p *= .029) as well as again a significant main effect of hemisphere (*F*[1, 19] = 6.356, *p *= .021) and interaction of hemisphere and group (*F*[1, 19] = 6.448, *p *= .020). Figure 4a shows the mean pattern MMN source waveforms for this grouping and for both hemispheres where the large overall difference of the groups as well as the strong lateralization in the correctly identifying subjects is quite obvious.

Four musicians and five nonmusicians reported that they had had the subjective impression of a musical meter or beat in the stimulation sequence. When subjects were grouped according to this criterion in the analysis (Figure 4b), the analysis of variance of the pattern MMN amplitudes also shows a main effect of hemisphere (*F*[1, 19] = 4.923, *p *= .039) but neither a significant main effect of group (*F*[1, 19] = 0.047, *p *= .830) nor an interaction (*F*[1, 19] = 2.598, *p *= .123).

## Discussion

During passive listening, subjects pre-attentively extracted a regular pattern from a very simple tone sequence. A prevailing pattern of three lower tones followed by a higher tone (AAAB) was embedded in a pseudorandom sequence of lower and higher tones. This AAAB pattern occurred with a rate of 0.5 within the stimulation sequence, whereas sequences of more than three lower tones in a row had lower probabilities (e.g., AAAAB with a rate of 0.25, AAAAAB with a rate of 0.125 and so on). A pattern MMN was elicited by tones that violated the predominant pattern, that is, by every fourth tone in a row (AAAA). The fact that the pattern MMN was significantly stronger in subjects who noticed the pattern is in line with previous results showing that the MMN is increased in subjects expressing explicit knowledge of the deviant stimuli [6]. However, a pattern MMN was also visible in subjects who did not express detection of the pattern, indicating that conscious recognition is not a precondition for the emergence of this pattern MMN. Apparently, the pattern can be extracted pre-attentively and violations can be detected even without conscious detection of the deviants. It should be noted that more musicians than nonmusicians reported pattern detection. Although a chi-square test yielded a non-significant result, probably due to a small sample size and corresponding low statistical power, the trend indicates that musical expertise might enhance this process.

In music theory, the grouping of strong and weak beats is summarized by the concept of meter. In our study, the B tone might be perceived as a strong beat and A tones might be perceived as weak beats, and thus the prevailing pattern (AAAB, or if slightly differently grouped: BAAA) could be interpreted as a Western 4/4 meter. Since the stimulation sequence in the present study might have been perceived as having a beat or meter, the pattern MMN might have occurred in response to violations of this meter. In line with this reasoning, our finding of a left-lateralization of the pattern MMN in musicians could be comparable to results of Vuust and colleagues [20] who found a strongly left-lateralized MMN in musicians in response to metrical violations in complex drum sequences. However, metrical saliency in our sequence was quite low, as we did not use samples of different drum instruments, and metrical complexity was much lower than in the study by Vuust et al. [20]. When subjects were asked if they perceived a beat or musical meter in the sequence, the number of subjects who reported this did not differ between musicians and nonmusicians. Also, when MMN amplitudes of all subjects were compared, no significant effect of meter perception was found. We thus conclude that the pattern MMN is not related to processing and perception of a musical meter but rather to the detection of a recurring pattern.

In contrast to previous studies on the pre-attentive encoding of metric regularities [20] and sequential patterns [3,14] in musicians and nonmusicians, in the present study the sequential regularity was encoded even in the absence of external cues like rhythmic context and without a pre-defined template of the standard pattern. That is, subjects extracted a pattern that was not explicitly marked, as it was, for example, in previous studies by van Zuijen et al [3,14] where the sequential pattern in the stimulus sequence was very transparent and easy to detect because of pitch jumps between consecutive patterns which marked the beginning and the end of one pattern sequence. In the present study the pattern did not 'pop out', but the pattern was instead embedded in a continuous stream of only two different tones. Whereas in most previous studies on the pattern MMN probabilities of deviants are around 0.1, here, the probability of a lower or higher tone after the mandatory three lower tones in a row was 0.5 each. Thus, the present results show that a pattern MMN can even be elicited under conditions where the pattern is violated as often as it is confirmed, that is, even with deviant probabilities as high as 0.5.

We presume that not the local conditional probability of a B tone appearing after three A tones (which was 0.5), but instead the global probability distribution of the possible patterns, with the AAAB pattern being by far the most frequent, induced the pattern MMN. It might be suggested that this pattern MMN could be based only on those instances where a pattern deviant sequence was directly preceded by at least two standard pattern sequences, which would establish the standard pattern within a local context. However, as the probability of consecutive standard-standard-deviant combinations within the whole stimulation sequence was only 0.125 we consider the local memory trace an insufficient explanation for the present result. In contrast, we suggest that the pattern MMN cannot only be based on a local memory trace but also on expectations or predictions generated from estimates of probabilities within a global context, that is, within the whole sequence. These global probabilities can only be extracted from long sequences of events which significantly exceed the length of sensory memory trace estimates that are found to be around 10 s [5]. For single tones, the influence of global probabilities on responses to oddball tones has been shown on the level of auditory cortex neurons in cats [21] as well as for the MMN in humans [12,13]. We now extend these findings and show that global probabilities of events affect processing of deviants not only on the level of single tones but also on a higher-order level of sequential patterns within tone sequences. This is in line with results of computer simulations showing that the cortical mechanisms underlying the MMN response might operate on several hierarchical levels, ranging from the level of single tones to complex sequential patterns [22]. With increasingly complex patterns, the time needed to extract enough regular instances of the pattern and build up a representation of the standard pattern also increases. Corresponding time constants of such higher-order pattern MMN in our data are unknown, and due to too little data points the development of the pattern MMN over time cannot be modeled. However, Ulanovsky and colleagues [21] estimated time constants of responses of auditory cortex neurons to single oddball tones, ranging from several hundreds of milliseconds for short-term effects of local probabilities up to several tens of seconds for long-term effects of global tone probabilities. For more complex tone patterns based on global probabilities, such as those used in our paradigm, we would expect even longer time constants.

The present results are interesting also from two other points of view, one regarding auditory grouping, and the other regarding the design of simple oddball experiments. In the present study, the *presence *of the pattern MMN can be seen as evidence that the AAAB sequence was encoded as a pattern. It can be assumed that these patterns were recognized through auditory grouping, as no other cue but the temporal order of the two different tones was available, similar to a study by Sussman and Gumenyuk [10] in which very similar sequential patterns were shown to induce auditory grouping. However, Sussman and Gumenyuk used a converse approach compared to ours: Using a completely regular sequence of consecutive AAAAB-patterns, they determined the stimulation rate at which the Bs did *not *elicit a pitch MMN any longer, arguing that only at this rate the AAAAB-pattern was encoded as an auditory object. Only at 200 ms SOA there was no (detectable) MMN to the B tones, whereas an MMN was still observed with 400 ms SOAs. We agree that the order of the tones in both studies induced auditory grouping of AAAB-patterns. However, the finding of the present pattern MMN seems to contradict the conclusion that such a pattern can only be pre-attentively grouped at very fast stimulation rates, as the present presentation rate of 1 s was far longer than the estimated limit of 400 ms in the study by Sussman and Gumenyuk [10]. We suggest that auditory grouping can also occur at much slower rates than previously suggested, and that responses to *violations *of patterns made up by auditory grouping might be a more sensitive measure to detect this.

The present results are also relevant regarding the design and analysis of oddball paradigms. Our results suggest that combinations of standards and deviants in very simple oddball sequences can be pre-attentively grouped and perceived as patterns, and that the probability distribution of the different possible patterns can induce the perception of a predominant pattern and evoke a corresponding pattern MMN. Therefore, tones that seem to be standards when regarded individually might actually function as pattern deviants when regarded in a more global context. This should be taken into account in the design and analysis of oddball sequences, because the amplitude of the MMN of interest (e.g., to a pitch deviation) might be diminished and its lateralization might possibly be biased if such pattern deviants are used as standards in the data analysis.

As expected, in the comparison of musicians and nonmusicians no group differences or interactions in the amplitude of the classic pitch MMN were found [as, for example, in [16]]. However, there was also no significant main effect between the groups of musicians and nonmusicians in the pattern MMN, indicating that musical training does not generally enhance the tendency to perceive patterns in the present paradigm. However, previous studies reported differences in pattern processing between musicians and nonmusicians. Musicians show a larger MMN in response to unexpected tones in mono- and polyphonic melodies [15,16]. In studies on more simple tone patterns, musicians show stronger pattern MMN in response to unexpected additional tones in ascending patterns, but groups do not differ for simple pitch patterns [3]. Also, musicians can better detect deviations from numerical regularities (a different number of tones within patterns of regular duration), but both musicians and nonmusicians can detect tones violating a temporal regularity (longer duration of the pattern with a regular number of tones) [14]. Although there were important differences to our paradigm (pattern probability, deviant probability, tone SOA), which were expected to make pattern extraction more difficult, the present stimulation sequence resembles the conditions of the aforementioned pattern MMN studies [3,14] in which no group differences were found. However, as there was a definite trend, a main effect of group might be observed with higher statistical power including more test subjects.

In any case, it should be noted that we did find differences in processing between groups in our study, since, as the interaction effect shows, musicians process the pattern violation more strongly in the left hemisphere than nonmusicians do. As more specialized abilities tend to have more strongly lateralized neuronal correlates in humans, the faculty of language being a prime example of this rule, it seems consistent that musicians might have developed a more strongly lateralized representation of the ability to temporally integrate and analyze tone sequences. In studies that used sequential patterns of simple tone stimuli similar to our stimulation sequence, albeit at a much faster stimulation rate, no lateralization of the MMN was found in nonmusicians or musicians [3,14]. This might be due to the fact that the used EEG technology in these studies as compared to MEG is less sensitive to detect lateralization effects.

Wolford and colleagues [23] propose that the generation of hypotheses and expectancies and the detection of event patterns in a probability guessing task is mainly supported by the left hemisphere. This might also be related to the observed left-lateralization of the MMN in response to expectancy violations in our study. The event frequencies and possible corresponding patterns in the study of Wolford and colleagues [23] only emerge from a longer sequence of events. This seems to be in line with our interpretation that the present, left-lateralized pattern MMN reflects violated expectancies generated from a global rather than local analysis of the tone sequence.

## Conclusion

In the present study a pattern MMN is observed in musicians and nonmusicians, which is based on auditory grouping of a sequential pattern (AAAB) and on the global context given by the probability distribution of possible patterns, rather than on a local memory trace. This pattern MMN is related to behavioural detection of the sequential pattern. The left-lateralization of the pattern MMN in musicians likely reflects higher specialization due to their long-term musical training.

## Methods

### Subjects

12 musicians and 12 nonmusicians participated in the experiment. Data of two musicians and one nonmusician had to be discarded due to insufficient data quality. Musicians (mean age = 25.6 years ± 3.4 SD, 5 male) were music students or young professionals who had received at least 10 years of musical training and were still actively playing their instruments. Nonmusicians (mean age = 27 years ± 2.4 SD, 5 male) had not received more than 2 years of musical training (and in most cases none at all) apart from compulsory musical education in school. All subjects were right-handed according to the Edinburgh Handedness Inventory [24] and had normal hearing as assessed by clinical audiometry. All subjects gave understanding and written consent to participate in the experiment and received monetary compensation. All procedures were carried out according to the declaration of Helsinki and were approved by the ethical committee of the medical faculty of the University of Muenster.

### Stimuli and Procedure

**Figure 5:**
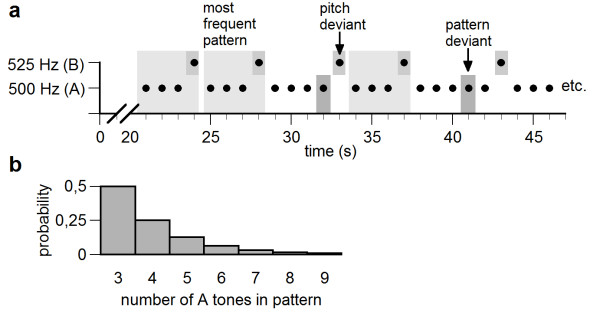
**Illustration of the design**. Illustration of a short part of the stimulation sequence including the most frequently occurring pattern of three tones followed by a higher tone (indicated by light grey boxes). Pitch and pattern deviants are indicated by medium and dark grey boxes, respectively (a). Illustration of the probability distribution of the possible patterns, which are defined by the number of A tones within the pattern (AAAB, AAAAB, etc.) (b).

Subjects passively listened to a continuous, pseudo-random 500 tone sequence composed of only two different sinusoidal tones, a lower 500 Hz (A) and a higher 525 Hz tone (B). Tones were of 400 ms length with 10 ms rise and decay time. Stimulus onset asynchronies of individual tones within the sequence varied randomly between 900 and 1100 ms. The order of tones within the stimulation sequence was pseudo-random and was governed by a higher-order regularity: Each high tone was preceded by at least three low tones (AAAB), whereby the probability of occurrence of longer sequences (e.g. AAAAB, AAAAAB and so on) decreased quadratically. As such, the AAAB pattern occurred with a rate of 0.5 within the stimulation sequence, AAAAB had a probability of 0.25, AAAAAB had a probability of 0.125 and so on. A short part of an exemplary stimulation and the overall probability distribution of possible patterns, defined by the number of A tones before a B tone, are depicted in Figure 5.

The AAAB sequence represented the most frequent sequence embedded in the continuous stimulation and can be considered a 'hidden' sequential pattern. With respect to this predominant pattern, every fourth low tone in a row which was not raised (AAAA) was a violation of this pattern and can be considered a 'pattern deviant'. We hypothesized that the pattern deviants would elicit an MMN response when compared to 'standard' low tones (AAAB) if the AAAB pattern was encoded as the predominant pattern. B tones (pitch deviants) were expected to elicit a pitch MMN, because they differ from A tones regarding their pitch, independent of their position in the sequence.

After the MEG measurements, subjects were asked if they had perceived a pattern or regularity within the stimulation sequence and if they could describe it, and also if they had perceived the sequence as having a musical meter or a 'beat'.

### Data acquisition and analysis

Magnetoencephalographic data were acquired with a 275 channel whole head system (Omega 275, CTF Systems Inc.) in a silent and magnetically shielded room. MEG was used rather than EEG because it is better suited to investigate lateralization effects of the MMN. Subjects were seated comfortably upright and their head position was fixed with pads. Stimuli were delivered via plastic tubes at 60 dB SL above the individual hearing level, which was determined with an accuracy of at least 5 dB for each ear. During auditory stimulation subjects watched a silent movie of their own choice. Subjects were instructed to pay no attention to the acoustic stimulation, and to move, blink and swallow as little as possible in order to minimize artifacts in the recorded MEG data.

For each individual subject, epochs of 1 s duration synchronized to stimulus onset, beginning 200 ms before and ending 800 ms after stimulus onset, were extracted from the continuous data set. Epochs containing signal amplitudes larger than 3 pT were considered to contain artifacts and were excluded from averaging. Standards, pitch deviants and pattern deviants were averaged separately. In order to obtain datasets containing the MMN responses to the pitch and the pattern deviations, averaged standard trials were subtracted from averaged pitch deviant or pattern deviant trials, respectively. Thus, the same standards were used in the computation of both kinds of MMN.

The equivalent current dipole model was applied to the averaged and subtracted datasets for each subject. Two spatiotemporal dipoles in a spherical volume conductor, one in each hemisphere, were fit simultaneously to the averaged evoked field of the pitch MMN. Only subjects with dipoles explaining at least 90% of the magnetic field variance were included in the analysis, which resulted in the exclusion of two musicians and one nonmusician. Dipole positions were fixed and the source space projection method [25,26] was applied, collapsing the 275 channel data to one source waveform for each dipole. The dipole locations and orientations of the pitch MMN were used to derive the source waveforms for the pitch as well as for the pattern MMN, as it was not possible to clearly identify a pattern MMN in the evoked fields of all subjects.

Amplitudes of the pitch and of the pattern MMN in the source wave forms were compared between groups and hemispheres in mixed model analyses of variance on the mean amplitudes within a 10 ms time window around the overall mean latency of the pitch and pattern MMN, respectively. According to subjects' responses to the questionnaire, further analyses of variance of the pattern MMN with different groupings were conducted to determine the influences of conscious awareness of the pattern and subjective impression of a musical meter on the physiological results.

## Authors' contributions

SCH, CL and CP designed the experiment and interpreted the results; SCH carried out data acquisition and analysis and drafted the manuscript; CL and CP revised the manuscript. All authors read and approved the final manuscript.
